# New threats in the recovery of large carnivores inhabiting human-modified landscapes: the case of the Cantabrian brown bear (*Ursus arctos*)

**DOI:** 10.1186/s13567-024-01279-w

**Published:** 2024-02-23

**Authors:** Ana Balseiro, Gloria Herrero-García, Juan Francisco García Marín, Ramón Balsera, Juana María Monasterio, David Cubero, Gabriel de Pedro, Álvaro Oleaga, Alberto García-Rodríguez, Israel Espinoza, Benjamín Rabanal, Gorka Aduriz, José Tuñón, Christian Gortázar, Luis José Royo

**Affiliations:** 1 Departamento de Sanidad Animal, Facultad de Veterinaria, Universidad de León, 24071 León, Spain; 2 Dirección General de Medio Natural y Planificación Rural del Principado de Asturias, 33007 Oviedo, Asturias Spain; 3 Dirección General de Patrimonio Natural y Política Forestal de La Junta de Castilla y León, 47014 Valladolid, Castilla y León Spain; 4 Sociedad de Servicios del Principado de Asturias S.A. (SERPA), La Laboral, 33203 Gijón, Spain; 5 Laboratorio de Técnicas Instrumentales, Facultad de Veterinaria, Universidad de León, 24071 León, Spain; 6 Departamento de Sanidad Animal, NEIKER-Instituto Vasco de Investigación y Desarrollo Agrario, 48160 Derio (Bizkaia), Spain; 7 Fundación Oso de Asturias, 33114 Proaza, Asturias Spain; 8 SaBio-IREC (CSIC-UCLM), 13071 Ciudad Real, Spain; 9 Departamento de Biología Funcional, Genética, Universidad de Oviedo, 33006 Oviedo, Spain

**Keywords:** brown bear, *Ursus arctos*, infectious diseases, traumas, wildlife mortality, pathology

## Abstract

Understanding mortality causes is important for the conservation of endangered species, especially in small and isolated populations inhabiting anthropized landscapes where both natural and human-caused mortality may hinder the conservation of these species. We investigated the mortality causes of 53 free-ranging brown bears (*Ursus arctos*) found dead between 1998 and 2023 in the Cantabrian Mountains (northwestern Spain), a highly human-modified region where bears are currently recovering after being critically threatened in the last century. We detected natural traumatic injuries in 52.63% and infectious diseases in 39.47% of the 38 bears for which the mortality causes were registered, with 21.05% of these cases presenting signs of both infectious diseases and traumas. More specifically, almost 30% of the bears died during or after intraspecific fights, including sexually selected infanticide (10.53%). In addition, primary infectious diseases such as infectious canine hepatitis, distemper, clostridiosis and colibacillosis caused the death of 15.79% of the bears. The number of direct human-caused deaths (i.e., shooting, poisoning, snare) decreased over the study period. This study also reveals three new mortality causes triggered by pathogens, two of which—*Clostridium novyi* and verotoxigenic *Escherichia coli*—not previously described in ursids, and the other one, canine distemper virus, never reported in brown bears as cause of death. New management strategies for the conservation of Cantabrian bears, which are urgently needed due to the rapid expansion of the population, should consider the mortality causes described in this study and must promote further research to elucidate how the high prevalence of infectious diseases may threaten the current recovery of the population.

**Supplementary Information:**

The online version contains supplementary material available at 10.1186/s13567-024-01279-w.

## Introduction

Worldwide human population has exponentially increased during the last century, leading to an intensification of activities of human origin (land use, exploitation of natural resources) that has shaped all type of ecosystems. Consequently, wildlife species are now forced to live in fragmented and anthropized landscapes, interacting more commonly with humans and their activities than ever before [1]. Many of the most threatened and isolated populations of large carnivores in Europe inhabit heavily humanized regions, which often promotes social conflicts that may hinder the current recovery of populations still relying on heavy conservation efforts and important economic investments [2]. Although environmental stochasticity and demography are usual constrains on the viability of small populations [3], deterministic density independent processes such as human-caused mortality may trigger a severe decline of these populations [4]. Therefore, the identification of the main mortality causes of endangered populations is needed not only for the detection and recognition of possible conservation problems or risks, but also for a correct design of conservation strategies and management programs. Not intentional but direct human-caused mortality (e.g., road-mortality) may be an important threat for the survival of endangered carnivores (e.g., the Iberian lynx *Lynx pardinus*) [5]. In addition, intentional illegal persecution (e.g., poaching, poison) still occurs in most European populations of large carnivores, including those critically endangered [6].

The brown bear is the largest living terrestrial carnivore. Although persecuted for centuries, most European populations have recently recovered. Although brown bears usually avoid areas with high human density, most European brown bear populations are currently inhabiting highly human-modified landscapes [2]. Current increasing brown bear numbers and the ubiquity of human activity in most bear areas in Europe generate constant interactions between bears and humans. These interactions, especially those concerning bear attacks to people and/or to human properties (e.g., beehives, livestock), complicate human-bear coexistence [7, 8], and may trigger an increase of human-related bear mortality, compromising the recovery of threatened and isolated brown bear populations.

Pathogens are natural components of ecosystems and contribute to modulating the population dynamics of all wild vertebrates. Pathogen emergence depends on interactions between host species and the environment. The extent of such interactions, and hence, pathogen emergence, is influenced by changes in climate, land use, and animal management [9]. Small and relatively isolated populations, such as those of brown bears and other large carnivores, are especially vulnerable to infectious disease outbreaks. Thus, diseases must be considered in the management of endangered species [10]. Recently, several emerging pathogens such as canine adenovirus type 1 (CAdV-1; the etiological agent of infectious canine hepatitis) or canine distemper virus (CDV; the etiological agent of distemper) have been detected in free-ranging brown bears in Eurasia and North America [11–14]. However, there is still insufficient knowledge on how these and other transmissible diseases may threaten the conservation of brown bear populations.

Here, we present an updated analysis of the mortality causes of the Cantabrian brown bear population, a small and isolated population currently recovering after being critically threatened in recent years. Specifically, we (1) analyze different mortality causes reported, (2) describe new mortality causes never previously described in free-ranging brown bears, (3) discuss how old and new threats may hinder the recovery of large carnivores, and (4) provide specific management and conservation recommendations that we believe may ensure both the current recovery of Cantabrian bears and the coexistence between bears and humans and their activities not only in the area but also in other highly human-modified landscapes.

## Materials and methods

### Study area and Cantabrian bear population

**Figure 1:**
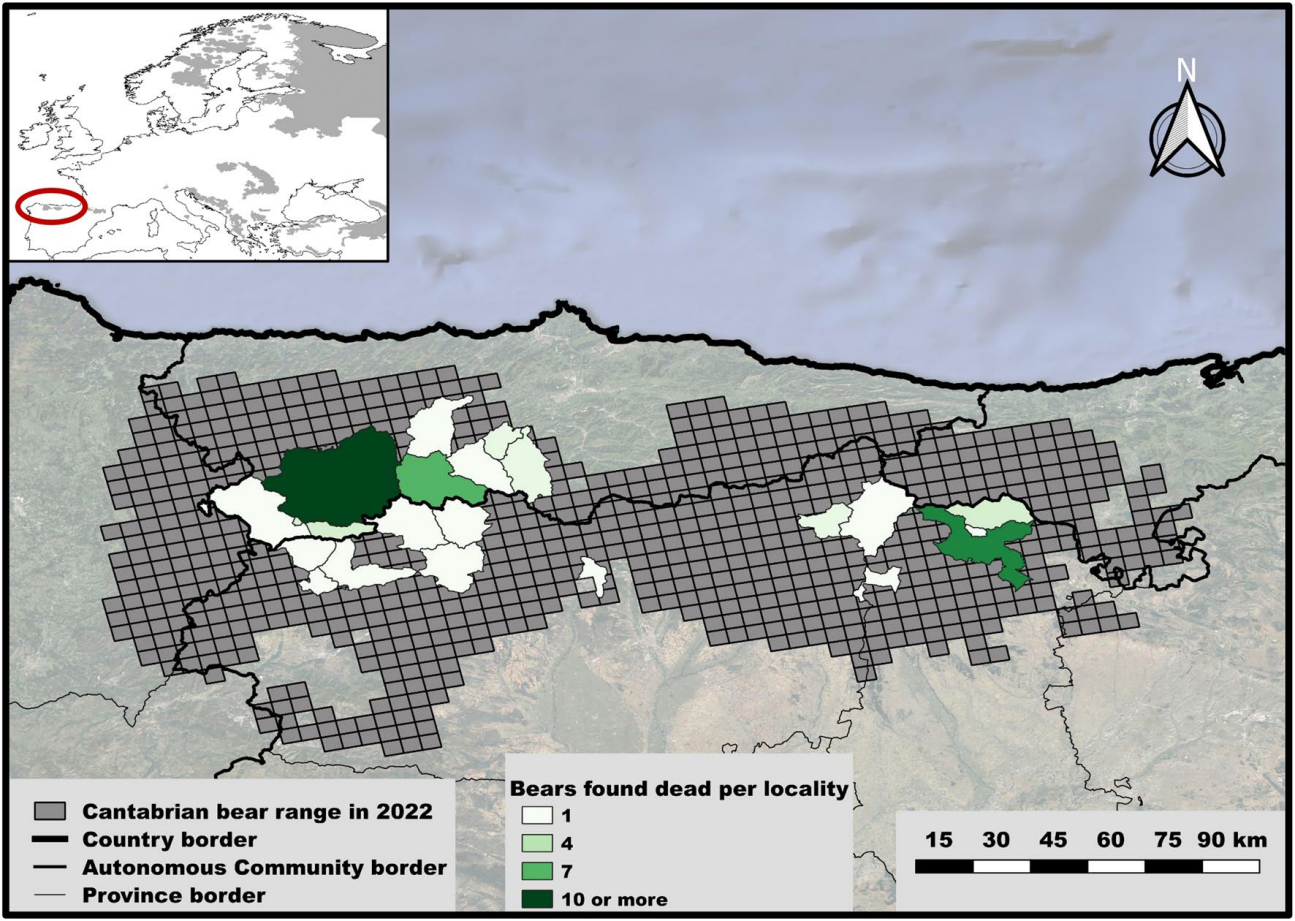
**Map indicating the number of Cantabrian brown bears found dead in each locality**. The current Cantabrian brown bear range as predicted in [18] is shown as grey grids. A map showing the entire brown bear distribution in Europe can be seen in the upper-left corner.

The brown bear population located in the Cantabrian Mountains (northwestern Iberian Peninsula) represents the westernmost population of the species in Europe (Fig. 1). Current Cantabrian bear distribution lies primarily in the temperate, broadleaf, and mixed forests biome, but some areas in the southern limit belong to the Mediterranean forests, woodlands and scrub biome [15]. The area ranges from 100 to 2648 m above sea level (m.a.s.l.) and annual mean precipitation fluctuates between 400 and 700 mm (mm) in the southern slopes and 900–1900 mm in the northern ones [16].

The landscape is dominated by mixed forests, meadows, rocky mountains, and small human settlements. Forests are primarily composed by different oak species such as *Quercus petraea*, *Q. pyrenaica*, *Q. orocantabrica*, or *Q. ilex* ssp*. rotundifolia*, beech *Fagus sylvatica*, chestnuts *Castanea sativa* and birch *Betula pubescens* [16]. Among wild mammals, wolves *Canis lupus*, several species of mesocarnivores (red foxes *Vulpes vulpes*, Eurasian badgers *Meles meles*, wildcats *Felis silvestris*, pine and stone martens *Martes* spp., etc.) and ungulates (red deer *Cervus elaphus*, roe deer *Capreolus capreolus*, Pyrenean chamois *Rupricapra pyrenaica* and wild boar *Sus scrofa*) are also present in the area.

Human population density is around 10.9 and 4.9 people/km^2^ for the western and eastern part of the mountain range, respectively [17]. Cattle raising represents the most important economic income for the region. Other human activities such as tourism (hiking, skiing, wildlife watching) are increasingly important in recent years. Mining activities have shaped the landscape for decades, but their importance is strongly declining.

Although almost extinct during the last century, primarily due to human persecution and habitat loss [18], Cantabrian bears have recovered from less than 100 individuals in the nineties to approximately 325 in the present [19, 20]. This recovery has been possible thanks to the legal protection of the population, which is strictly protected since 1973, currently catalogued as Endangered by the Spanish Inventory of Endangered Species and included in the Annexes II and IV of the Habitats Directive 92/43/EEC [21]. Nowadays, Cantabrian bears are present in around 18 000 km^2^ and in four different administrative regions (Autonomous Communities) of Spain: Galicia, Asturias, Castilla y León, and Cantabria [18].

### Field data collection: location of dead bears and exploratory analysis of the area

Fifty-three free-ranging Cantabrian brown bears: 33 subadults or adults (i.e., animals older than two years; “adults” hereafter), 16 cubs (including both cubs of the year and yearlings up to two years; “cubs” hereafter) and 4 undetermined due to insufficient tissue availability; 28 males, 12 females and 13 undetermined, found dead in Asturias (*n* = 29) and Castilla y León (*n* = 24) were necropsied between 1998 and 2023 (detailed information in Table 1).

**Table 1:** **Available data, cause of death, pathological findings, and classification of death of fifty-three free-ranging Eurasian brown bears (*Ursus arctos*) necropsied from 1998 to 2023 in Asturias and Castilla y León (northwestern Spain)**.

Bear	Date	Age	Sex	Cause of death	Classification of death
1	08/05/1998	Adult (7 years)	Male	Snare/exertional myopathy/gangrenous myositis (*Clostridium sordellii* and *C. bifermentans*)	H/I/NT
2	12/06/1998	Cub	Female	Infanticide	NH/NI/T
3	10/06/2000	Adult	N.d	N.d	–
4	06/06/2005	Adult	Male	N.d	–
5	26/09/2005	Adult	Male	Intentional shooting	H/NI/NT
6	19/11/2005	Adult	N.d	Poisoning: strychnine	H/NI/NT
7	14/06/2008	Cub (1 year)	Male	Infanticide	NH/NI/T
8	27/08/2012	Adult	Male	Snare/gangrenous myositis (*C.sordellii*)	H/I/NT
9	29/10/2012	Cub (9 months)	Female	Died after handling and transport/exertional myopathy	H/NI/NT
10	12/06/2014	Adult (3 years)	Male	Fighting/gangrenous myositis (*C. sordellii* and *C. septicum*)	NH/I/T
11	15/06/2014	Adult (5 years)	Male	Infectious canine hepatitis	NH/I*/NT
12	12/12/2014	Adult (9 years)	Male	Fighting/septicemia	NH/I/T
13	29/04/2015	Adult (20 years)	Female	Neoplasia: cholangiocarcinoma	NH/NI/NT
14	23/05/2015	Cub (4 months)	Male	Infectious canine hepatitis	NH/I*/NT
15	16/10/2015	Adult	Male	Traumatic lesions/gangrenous myositis	NH/I/T
16	05/03/2016	Adult	Male	Traumatic lesions due to fall	NH/NI/T
17	08/10/2016	Adult	Male	Intentional shooting	H/NI/NT
18	27/11/2016	Adult (6 years)	Female	Snare/strangled	H/NI/NT
19	07/01/2017	Adult (6 years)	Male	Mushroom poisoning; hepatic and renal necrosis	NH/NI/NT
20	02/04/2017	Cub (3 months)	Female	Infectious canine hepatitis	NH/I*/NT
21	21/04/2017	Adult (19 years)	Male	Fighting and cliff fall	NH/NI/T
22	21/04/2017	Adult (20 years)	Male	Fighting and cliff fall	NH/NI/T
23	29/09/2018	Adult (4 years)	Female	Traumatic lesions/gangrenous myositis (*C. sordellii*)	NH/I/T
24	27/10/2018	Adult (5 years)	N.d	N.d	–
25	08/11/2018	Adult (7 years)	Male	N.d	–
26	21/09/2019	Cub (1 year)	N.d	N.d	–
27	13/12/2019	Cub (1 year)	Male	N.d	–
28	06/04/2020	Adult	N.d	N.d	–
29	23/06/2020	N.d.	N.d	N.d	–
30	01/09/2020	N.d.	N.d	N.d	–
31	23/09/2020	Cub	Female	Traffic accident	H/NI/NT
32	29/11/2020	Adult	Female	Accidental shooting	H/NI/NT
33	02/05/2021	N.d.	N.d	N.d	–
34	04/06/2021	Cub (1 year)	Male	Craneoencephalic traumatism/infanticide	NH/NI/T
35	09/06/2021	Cub (6 months)	Male	Craneoencephalic traumatism/infanticide	NH/NI/T
36	15/06/2021	Adult (2 years)	Male	Craneoencephalic traumatism/ethylene glycol intoxication	H/NI/T
37	24/06/2021	Adult (2 years)	Male	Traumatic lesions	NH/NI/T
38	17/07/2021	Cub (7 months)	Female	Traumatic lesions	NH/NI/T
39	20/09/2021	N.d	N.d	N.d	–
40	06/05/2022	Adult (8 years)	Male	Traumatic lesions/gangrenous myositis (*Clostridium novyi*)	NH/I/T
41	06/06/2022	Adult (8 years)	Female	Traumatic lesions/toxemia and septicemia (verotoxigenic *Escherichia coli*)	NH/I/T
42	06/06/2022	Adult	Male	Traumatic lesions/cliff fall	NH/NI/T
43	23/06/2022	Adult	Male	Traumatic lesions/fighting and cliff fall	NH/NI/T
44	03/09/2022	Adult	Female	N.d	–
45	03/09/2022	Cub	N.d	N.d	–
46	03/09/2022	Cub	N.d	N.d	–
47	26/09/2022	Cub (8 months)	Female	Craneoencephalic traumatism/distemper	NH/I*/T
48	26/01/2023	Cub (3 weeks)	Male	Colibacillosis/*Escherichia coli*	NH/I*/NT
49	22/05/2023	2 years	N.d	Traumatic lesions/gangrenous myositis (*Clostridium novyi*)	NH/I/T
50	29/05/2023	Adult	N.d	N.d	–
51	18/08/2023	Cub (8 months)	Male	Traffic accident	H/NI/NT
52	28/08/2023	Adult	Male	Traffic accident	H/NI/NT
53	02/09/2023	12 years	Male	Traffic accident/gangrenous myositis (*C. sordellii*)	H/I*/NT

*N.d.* not determined, *H* direct human-intervention, *NH* non-direct human intervention, *I* infectious disease, *NI* non-infectious disease, *T* traumatic injury due to infanticide, fighting or falling, *NT* non-traumatic injury. Bears 5, 7, 9, 11, 12, 15, 18, 24, 25, 28, 29, 30, 31, 32, 33, 39, 42, 43, 44, 45, 46, 50, 51 and 52 were found in Castilla y León and the remaining ones in Asturias. An asterisk (*) indicates primary infectious disease.

Bear carcasses were found opportunistically, either during field inspections by rangers working on the conservation of the species, or by tourists or villagers. The surroundings of each carcass location were inspected to look for evidence about the most possible mortality cause. Each bear was brought to a center specialized on post-mortem examinations (the list of the centers is specified in section “Laboratory data collection: brown bear necropsies and diagnostic procedures”) and preserved at 4 °C for a maximum of 24–48 h before we conducted complete post-mortem examinations of each individual. As shown in Table 1, the number of necropsies has increased during the last decade probably due to the population recovery.

### Laboratory data collection: brown bear necropsies and diagnostic procedures

We performed the necropsies of Cantabrian bears in four centers specialized on post-mortem examinations: (1) the University of León, (2) the Regional Service for Research and Agri-food Development of Asturias (SERIDA), and the Wildlife Rehabilitation Centers of (3) Castilla y León and (4) Asturias. We took samples of several tissues for evaluation (encephalon, spinal cord, tongue, heart and skeletal muscle, lungs, liver, gallbladder, kidneys, adrenal glands, urinary bladder, spleen, pancreas, gut, genital tract, and lymph nodes) and used standard methods in histopathology, immunohistochemistry [13], microbiology, and toxicology [12, 22, 23]. Histopathological studies were systematically performed in all necropsied bears; whereas immunohistochemical, microbiological or toxicological studies were performed to confirm the diagnostic if either macroscopic or histological studies suggested that death was due to a specific infectious disease or toxic. Each method is described in detail in the following sections. Histological stains included hematoxylin–eosin, Gram, Ziehl–Neelsen, Von-Kossa, Mallory Azan, Klüber-Barrera and Masson’s trichrome. Whenever possible, we performed a dental histological study of the first molar or canine tooth to determine the age of each bear [24]. Toxicology was performed by chromatography in AMS lab (a private company located in Lugo, Spain) or in SaBio-IREC (Ciudad Real, Spain). We considered necropsy findings, laboratory results and characteristics of the carcass location site to draw conclusions about the most possible cause of death of each bear, classifying mortality causes according to three different and non-mutually exclusive criteria: (1) human origin (whether the cause of death was related to any direct human activity such as snare, shooting, poisoning or traffic accident; yes/no), (2) trauma (whether each bear died due to a natural traumatic injury such as infanticide, fighting or falling; yes/no), and (3) infectious disease (whether an infectious disease was found in each individual; yes/no). We classified each infectious disease as primary (if the disorder was not associated with or caused by a previous disease or trauma) or as secondary (if the disorder was associated with a previous disease or trauma).

### Clostridial isolation

We placed skeletal muscle samples of bears 1, 8, 10, 23, 40, 49 and 53 on 5% sheep blood agar plates and incubated them at 37 °C for 24–48 h in an anaerobic box with an atmosphere generator (GENbox anaer; bio-Mérieux, France). We sub-cultured in the same growth medium those colonies showing typical morphology of the main histotoxic clostridia (Table 2) [25] and identified them by conventional methods, such as Gram stain and Vitek2system’s AN card (bio-Mérieux, France).

**Table 2:** **Main characteristics of the major histotoxic clostridial pathogens of animals **[25]

Species	*Clostridium* spp. cultural characteristics			
	Colony Diameter (mm)	Hemolysis	Colony Margin	Other characteristics
*C. chauvoei*	0,5–3	β-hemolytic		
*C. novyi*	1–5	β-hemolytic	Scalloped or rhizoid margin	Circular or irregular colonies
*C. septicum*	1–5	β-hemolytic	Irregular edges	May swarm over agar
*C. sordellii*	1–4	Slightly β-hemolytic	Variable	Circular or irregular colonies

### qPCR pathogen detection and characterization

We isolated DNA either from (1) fresh liver and brain samples stored at 4 °C or from (2) paraffin embedded intestine, liver and adrenal gland samples. We used the Speedtools Tissue DNA extraction kit (Biottols, Madrid, Spain) or the NucleoSpin^®^ FFPE DNA kit (Macherey–Nagel, Bethlehem, USA) in each case, respectively.

We used the DNA isolated from embedded paraffin intestine, liver and adrenal gland to identify verotoxigenic (Shiga toxin producer) *Escherichia coli* (VTEC) in bears 41 and 48. More specifically, we perform qPCR using the Quantimix easy Probes Kit (Biotools, Madrid, Spain) in a StepOne Plus (Life technologies, Carlsbad, USA) and following the *Escherichia coli* VTEC screening kit PATHfinder (Generon, San Prospero (MO), Italy).

We tested DNA samples from fresh liver and brain for CAdV-1 detection in all bears when tissues were available. The procedure included two different quantitative polymerase chain reaction (qPCR) protocols. First, we amplified a 160-base pair (bp) long fragment of the ZFX gen of brown bear DNA [26], which allowed us to ensure DNA extraction and the absence of PCR inhibitors, and thus avoid false negatives. Second, we amplified a 160 bp long fragment comprising part of the E3 and U-exon genes of CAdV [27]. We used the Quantimix easy Kit (Biotools, Madrid, Spain) in a StepOne Plus Real-Time PCR System (Life technologies, Carlsbad, USA) in both qPCR. We used Cantabrian brown bear liver DNA sample and CAdV-1 extract DNA as positive controls, respectively.

For CDV identification we used the RNA isolated from embedded paraffin kidney and adrenal gland of bear 47. RNA was extracted using the Speedtools Total RNA extraction kit (Biottols, Madrid, Spain). qPCR was performed using the Canine Distemper Virus kit (Genesig, Primer Design, Camberley, UK) and High ScripTools-QUANTIMIX Easy Probes Master Mix (Biotools, Madrid, Spain) for reverse-transcription and qPCR, in a StepOnePlus Real-Time PCR System (Thermofisher Scientific,Paisley, UK) using positive and negative controls included in the CDV diagnostic kit.

### Immunohistochemistry

To detect *E. coli*, we prepared serial paraffin-embedded sections (3 µm) from tissue samples of brown bears 41 and 48 and stained them with primary polyclonal antibody against all O and K antigenic serotypes of *E. coli* (MyBiosource, San Diego, CA, USA) diluted 1:200 in TBS + BSA 0.1%. To detect microglia cells, we prepared serial paraffin-embedded sections (3 µm) from the central nervous system (CNS) samples of brown bear 47 and stained them with primary polyclonal antibody against antigen ionized calcium-binding adaptor molecule 1 (Iba1, Wako Chemicals Europe GmbH, Neuss, Germany) diluted 1:1000 in TBS + BSA 0.1%. Tissues from brown bear 47 were also stained with a commercial monoclonal antibody (mouse anti-raccoon dog CDV monoclonal antibody, clone DV2-12, MyBiosource, San Diego, CA, USA) diluted 1:1500 in TBS containing 0.1% BSA. We deparaffinized the sections and blocked any endogenous peroxidase activity by incubating them with 0.5% H_2_O_2_ in distilled water for 30 min. To prevent non-specific binding, we retrieved antigens (citrate pH 6.0 in microwave 20 min) and incubated the samples during 20 min in a humidified chamber with 5% goat (for polyclonal antibodies) or horse (for monoclonal antibody) normal serum and 0.1% bovine serum albumin in Tris-buffered saline. We then incubated tissue sections overnight at 4 °C in a humidified chamber with commercial polyclonal or monoclonal antibody diluted in TBS + BSA 0.1%. We washed the slides with TBS 1 × and incubated them with an anti-rabbit (Vector Laboratories, California, USA; for polyclonal antibodies) or anti-mouse (Vector Laboratories, California, USA; for monoclonal antibody) secondary antibody, diluted 1:200 in TBS + BSA 0.1%. We then incubated the samples for 30 min with the Avidin–biotin-peroxidase complex reagent-method (ABC Standard, Vector Laboratories, California, USA) in TBS 1x. We used NovaRed (Vector Laboratories, California, USA) as chromogen substrate to visualize labeling. Afterwards, we counterstained the slices with Mayer’s hematoxylin, dehydrated and mounted them with DPX (Fluka, Sigma, St. Louis, MO, USA). We used slices without primary antibodies and with intestine tissue from an *E. coli*-infected calf as negative and positive control, respectively. In addition, we used stained lymph node tissue and CNS from an infected badger for Iba-1 and CDV positive controls, respectively.

## Results

### Overview

**Figure 2:**
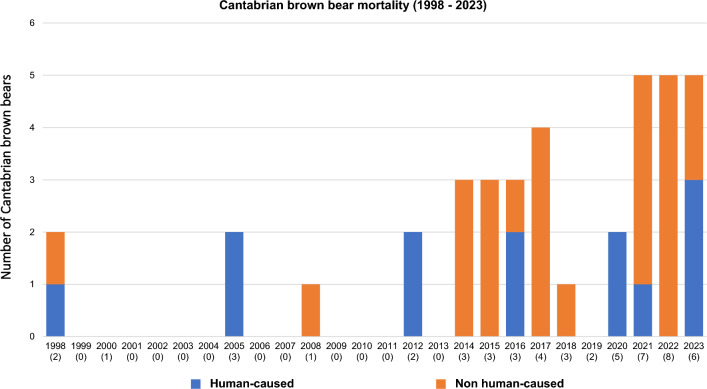
**Brown bears found dead in the Cantabrian Mountains (NW Spain) between 1998 and 2023 whose mortality cause could be inferred in this study**. Blue and orange areas in the Y axis indicate the number of brown bear deaths for which human activities were associated or discarded, respectively. Numbers in brackets in the X axis indicate the total number of brown bears found death each year, including those for which the ultimate cause of death could not be determined.

**Figure 3:**
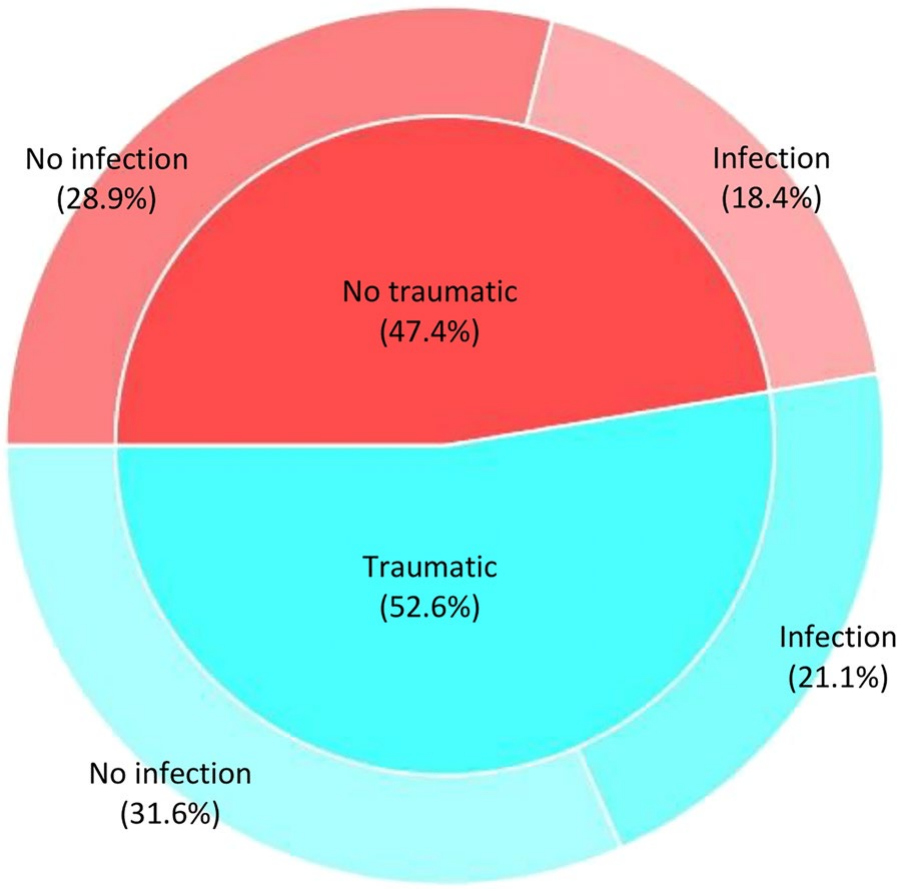
**Percentage of natural traumatic injuries (inner pie) and infectious diseases (outer doughnut) detected among the 38 Cantabrian brown bears whose mortality cause could be inferred in this study.**

**Figure 4:**
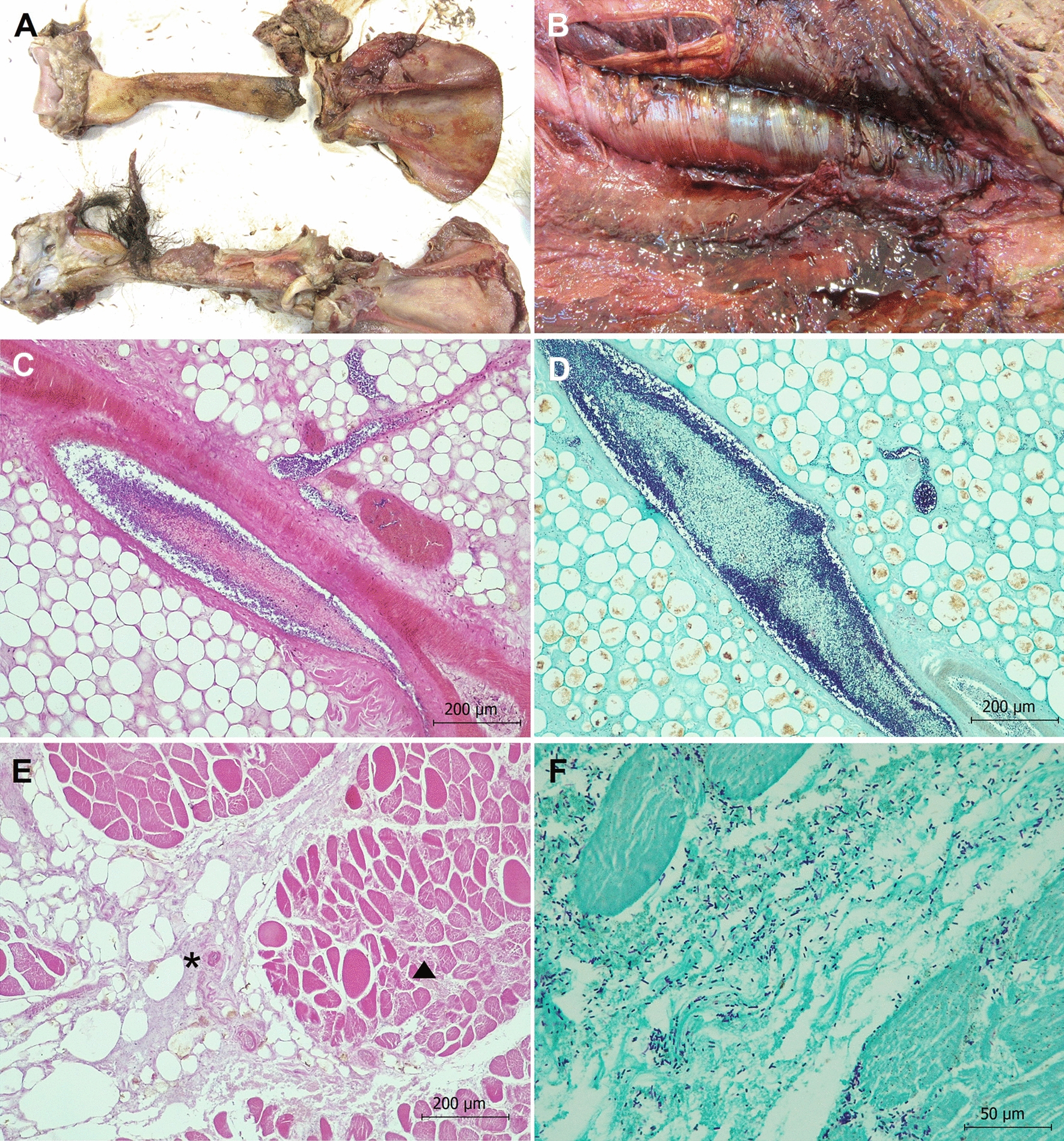
**Pathological findings in brown bear 40 showing clostridial gangrenous myositis due to *Clostridium novyi*.** **A** Humerus. Fractures of the head of the left humerus are shown. **B** Skeletal muscle. Serohemorrhagic edema, emphysema and hemorrhages are observed in skeletal muscles, mainly affecting the longissimus dorsi and iliocostalis lumborum. **C** Adipose tissue. Vascular damage with loss of endothelial cells and thrombus formed by clostridial-like bacilli are observed in the lumen of the vessels. Hematoxylin and eosin stain. **D** Adipose tissue. Thrombi formed by Gram positive clostridial-like bacilli are observed in the lumen of the vessels. Note that bacteria are invading the endothelium. Gram stain. **E**) Iliocostalis lumborum skeletal muscle. Hyperacute myodegeneration consisted of myonecrosis (arrowhead), edema, gas (asterisk), extravasation of fibrin into the interstitial spaces and lacunar dissolution of myofibers can be observed. Hematoxylin and eosin stain. F) Iliocostalis lumborum skeletal muscle. Numerous Gram positive clostridial-like bacilli are present, mainly located in the interstitial space. Gram stain.

**Figure 5:**
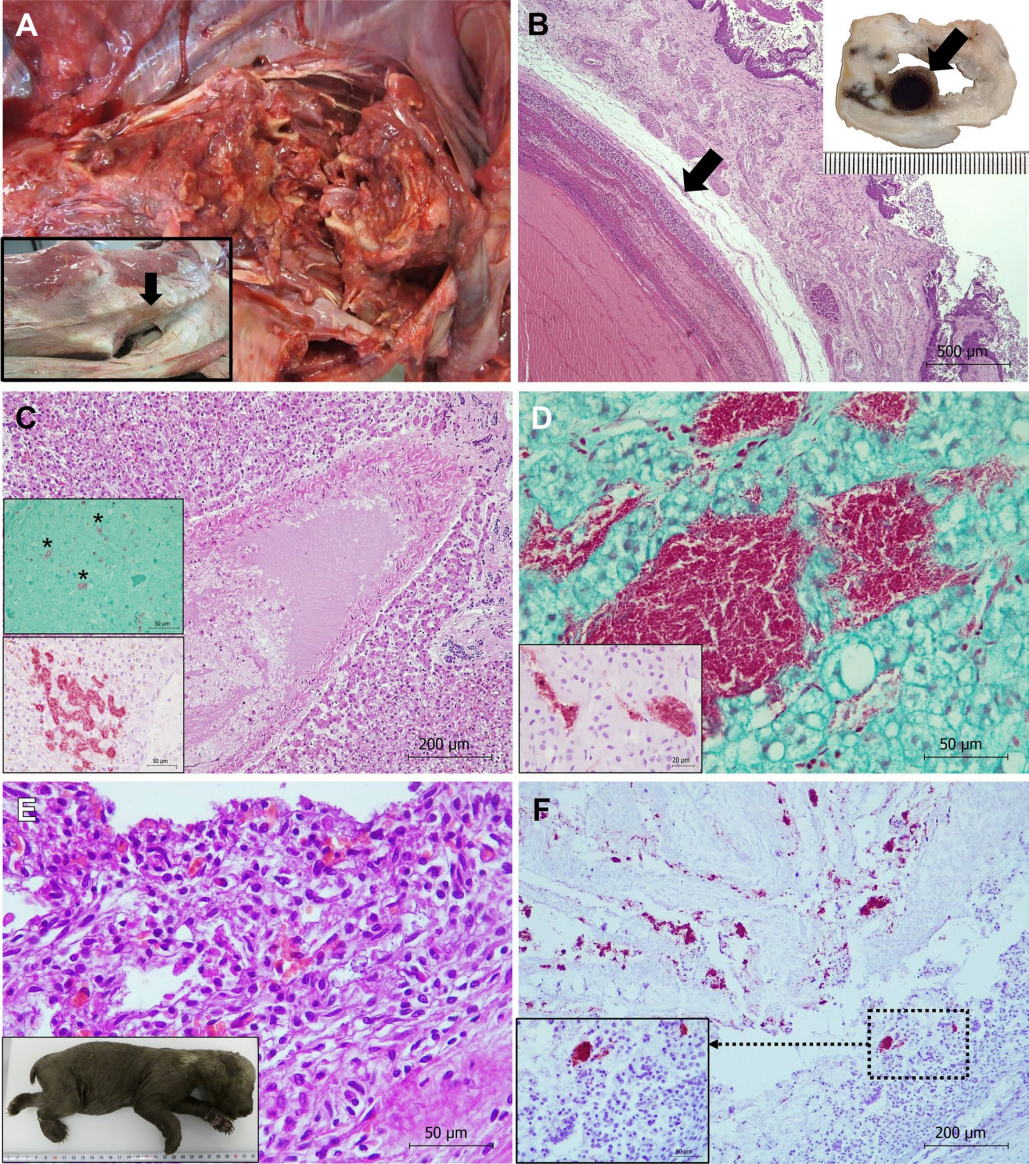
**Pathological findings in brown bears with colibacillosis**. **A**–**D**: Bear 41 (verotoxigenic *Escherichia coli* strain), **E**–**F**: Bear 48. **A** Vertebral column. Severe lesion located in L1-L2 with associated lordosis and lumbar scoliosis (inset, arrow). Osteolysis, loss of the intervertebral disc and spinal cord integrity, and presence of purulent material are also observed. **B** Cardia. Microscopic detail of the thrombus shown in inset (arrow). Loss of endothelial cells, hyaline degeneration of the vessel artery wall, and lines of Zahn are observed. Hematoxylin and eosin stain. Inset: One-centimeter thrombotic vascular lesion is observed (arrow). **C** Liver. A vascular thrombus is observed. Hematoxylin and eosin stain. Upper inset: Gram negative bacilli (asterisks) are present in the lumen of the thrombus. Gram stain. Lower inset: Numerous bacteria identified as *E. coli* using immunohistochemistry are present within hepatic sinusoids. ABC complex. **D** Adrenal gland. Presence of numerous Gram-negative bacilli inside blood vessels located in the adrenal cortex in the zona reticularis. Gram stain. Inset: Bacteria are identified as *E. coli* using immunohistochemistry. ABC complex. **E** Intestine. Presence of catarrhal enteritis with destruction of the intestinal microvilli of the intestinal mucosa. Inset: cub 48. **F** Numerous clumps of *E. coli* are identified in the intestinal lumen using immunohistochemistry. Avidin biotin complex.

**Figure 6:**
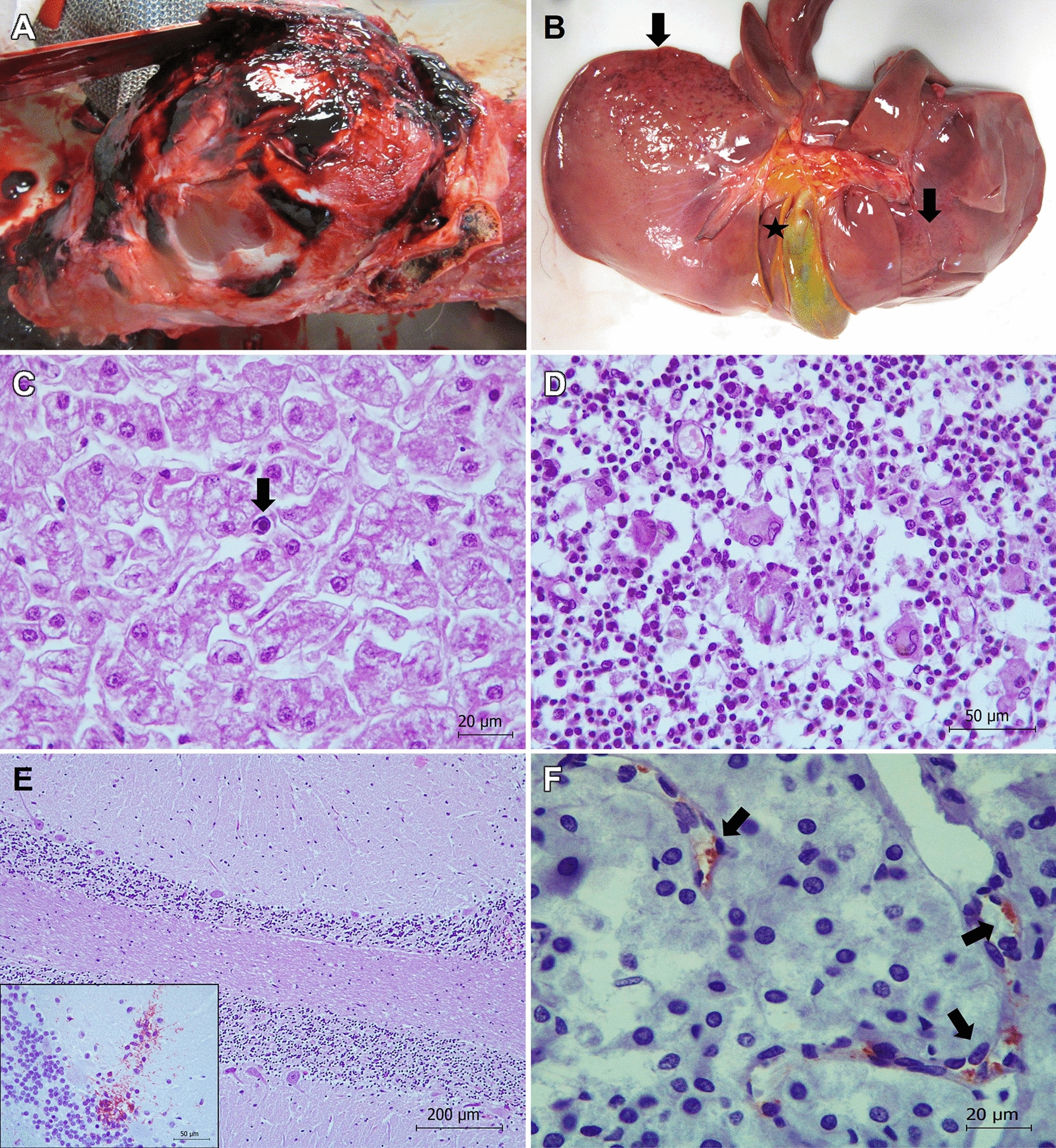
**Pathological findings in brown bear 47 showing distemper.** **A** Head. Subcutaneous and muscular hematomas and fracture of the parietal bone can be observed. **B** Liver. Petechial hemorrhages (arrows) and edema of the wall of gall bladder (asterisk) are observed in visceral surface. **C** Liver. Necrosis of hepatocytes and presence of a basophilic intranuclear inclusion body (arrow), without associated inflammatory infiltration, are shown. Hematoxylin and eosin stain. **D** Lymph node. Many syncytia formation (multinucleated giant cells) located in the cortical region are observed. Phenomena of karyorrhexis and karyolysis in several lymphocytes can be also observed. Hematoxylin and eosin stain. **E** Brain: cerebellum. Widespread necrosis and depletion of Purkinje cells in two cerebellar folia is shown. Hematoxylin and eosin stain. Inset: Proliferation of Bergmann glial cells is revealed using immunohistochemistry. Avidin biotin complex, Iba1 primary antibody. **F** Adrenal gland: canine distemper virus (CDV) antigen is detected within endothelial cells of blood capillaries located in medulla using immunohistochemistry. Avidin biotin complex, anti-CDV primary antibody.

We were able to infer likely mortality causes of 38 out of the 53 bears necropsied (71.7%, Table 1). Inadequate preservation of collected specimens and insufficient tissue availability mainly due to scavenging prevented the determination of the mortality causes of the remaining 15 individuals. We detected natural traumatic injuries in 52.63% and infectious diseases in 39.47% of the bears for which the mortality causes were registered, with 21.05% of these cases presenting signs of both infectious diseases and traumas (Tables 1 and 3; Figs. 2, 3). More specifically, almost 30% of the bears died during or after intraspecific fights, including sexually selected infanticide (10.53%). The relative importance of direct human-caused mortality decreased over the study period, and causes included snares, strychnine poisoning, ethylene glycol toxicosis (Additional file 1), shooting and traffic accidents (Table 1 and 3; Fig. 2). In addition, primary infectious diseases caused the death of 15.79% of the bears (Table 1). Regarding the 15 infectious diseases registered, 40% (6/15) were primary and 60% (9/15) secondary to traumas (either natural or human-caused). We detected bacterial and viral infectious diseases in eleven (73.33%) and four (26.67%) of the cases, respectively (Table 1). The prevalence of primary infectious diseases increased during the last decade (Table 1). In this context, in 2022–23 we detected three new mortality causes originated by two bacterial pathogens which had never been described in the Ursidae family (*Clostridium novyi* and verotoxigenic *Escherichia coli* VTEC; Figs. 4, 5), and by one virus not previously described as cause of death in free-ranging brown bears (CDV; Fig. 6). In addition, we found three individuals with disease caused by CAdV-1 (Table 1) [12]. Detailed information about the necropsies performed to each of the bears in which we found infectious diseases never previously reported in free-ranging Eurasian brown bears (i.e., gangrenous myositis due to *Clostridium novyi*, toxemia and septicemia due to VTEC, and CDV, Figs. 4, 5, 6) is provided in section “Infectious diseases detected for the first time in Eurasian brown bears”.

**Table 3:** **Percentage of human-caused mortality, infectious diseases and natural traumatic injuries found in free-ranging Cantabrian brown bears necropsied between 1998 and 2023.**

Period		Bears	Mortality causes of Cantabrian brown bears (non-mutually exclusive)				
			Human-caused %		Infectious disease %		Traumatic injury %
1998–2023	Cubs	12	25		33.33		50
	Adults	26	42.31		42.31		53.85
	Total	38	36.84		39.47		52.63

Please, note that mortality causes are not mutually exclusive

### Infectious diseases detected for the first time in Eurasian brown bears

#### *Gangrenous myositis due to Clostridium novyi (*Fig. 4*, Bears 40 and 49)*

External examination of bear 40 showed fractures in the head of the left humerus and in the body of the right ischium (Fig. 4A), hemorrhagic enteritis and acute gangrenous myositis as the main lesions present. Skeletal muscles, especially the longissimus dorsi (Fig. 4B), left humeral, scapular, and femoral muscles presented subcutaneous serohemorrhagic edema, and large hemorrhages and emphysema. Alveolar edema and congestion were observed in the lungs in the thoracic cavity, and hemoperitoneum, and congestion and loss of consistency in liver, kidneys, and spleen in the abdominal cavity. Microscopically, the main lesions were observed in the skeletal muscle, stomach, and small and large intestine. Vascular damage with loss of endothelial cells and thrombus formed by Gram positive clostridial-like bacilli could be observed in the lumen of the vessels in the adipose tissue (Fig. 4C, D), skeletal muscles, cardiac muscle, lungs, liver, kidneys, spleen and smooth muscle of stomach and intestine. Skeletal muscles additionally presented hyperacute myodegeneration consisting of myonecrosis, edema, gas, extravasation of fibrin into the interstitial spaces and lacunar dissolution of myofibers (Fig. 4E). Hemorrhagic enteritis affected the small and large intestine primarily. Gram staining showed Gram positive clostridial-like bacilli, most numerous in skeletal muscle (Fig. 4F), stomach and intestine. No relevant lesions were observed in other tissues. Clostridia isolated from skeletal muscle were identified as *C. novyi*.

In bear 49 only the skeletal bones, the skin and the longissimus dorsi skeletal muscle were found in the field. The animal showed ante mortem fractures of left ribs from the 7^th^ to the 13^th^ (Additional file 2), as well as fracture of the 9^th^ vertebral body. Histologically hemorrhage and presence of clostridia were observed at the level of the ribs and vertebral body fracture (Additional file 2). In addition, acute gangrenous myositis was diagnosed in longissimus dorsi muscle (Additional file 2), along with vascular damage and thrombus formed by Gram positive clostridial-like bacilli in the lumen of vessels. The same bacteria were also present within the interstitial spaces between myofibers (Additional file 2). Clostridia isolated from skeletal muscle were identified as *C. novyi*.

#### *Toxemia and septicemia due to Escherichia coli (*Fig. 5*, Bears 41 and 48)*

Macroscopically, bear 41 showed subcutaneous edema, and a severe lesion in the vertebral column (location L1-L2) with associated lordosis and lumbar scoliosis (Fig. 5A). It also showed osteolysis, loss of the intervertebral disc and presence of purulent material (Fig. 5A). We also detected a substantial loss of spinal cord integrity. Microscopically, the individual presented osteitis and associated purulent myositis consisted of muscle fiber atrophy, multifocal calcification foci and abundant connective tissue, which suggested an injury older than 15 days. The bear also showed a 1 cm nodular lesion in the cardia (Fig. 5B), that histologically corresponded to a thrombotic vascular lesion (Fig. 5B). A loss of endothelial cells and hyaline degeneration of the vessel wall, as well as the presence of Gram-negative bacilli adjacent to the tunica intima or in the lumen of the thrombus characterized the thrombotic vascular lesion. Thrombi with similar characteristics were also reported in arteries and veins from other locations, mainly in the liver (in the branches of the hepatic artery and portal vein, Fig. 5C) and in the right adrenal gland (inside blood vessels located in the adrenal medulla, Fig. 5D), but also in the skeletal muscle, lung, spleen, ovaries and intestine. The small intestine presented catarrhal enteritis with microvilli destruction in the intestinal mucosa. We also observed abundant fibrin forming networks both inside the crypts of Lieberkühn and in the lumen of the intestine. Numerous Gram-negative bacilli were present adhered to the surface of enterocytes. We observed congestion in all the organs mentioned and in the cardiac muscle. Lungs presented alveolar edema, while disseminated intravascular coagulation was observed in pulmonary arteries and veins. Hemorrhages, cholangitis and cholangiectasis were observed in the liver, with parasites being present inside the lumen of the interlobular bile ducts. We did not observe any other relevant lesion in other tissues. Immunohistochemistry confirmed that Gram negative bacteria observed in tissues were *E. coli* (Fig. 5C, D). qPCR molecular study identified VTEC positive in adrenal gland, liver and intestine samples.

In bear 48 catarrhal enteritis with microvilli destruction in the intestinal mucosa, and presence of numerous Gram-negative bacilli identified as *E. coli* by immunohistochemistry were observed (Fig. 5E, F). Hemorrhages in lungs and kidney and congestion in several organs were observed, as well as disseminated intravascular coagulation, mainly in the liver vessels. qPCR from paraffin embedded samples could not identify the *E. coli* strain.

#### *Distemper (*Fig. 6*, Bear 47)*

Bear 47 showed a cranioencephalic traumatism with fracture of the parietal bone (Fig. 6A). Gross lesions consisted of petechial hemorrhages in the liver (Fig. 6B), thymus, and heart, as well as thickening and edema of the gall bladder’s wall and congestion of spleen, kidney, and meninges (Fig. 6A, B). Microscopically the main pathological findings appeared in the liver, lymph nodes, brain, and kidney. The liver additionally showed hemorrhages, diffuse (panlobulillar) degeneration and necrosis of hepatocytes. Karyorrhexis and karyolysis and presence of basophilic intranuclear inclusions bodies were also detected in hepatocytes, but associated inflammatory infiltration was absent (Fig. 6C). The gall bladder showed edema of the wall. Congestion, hemorrhages, lymphoid depletion, karyorrhexis and karyolysis phenomena in lymphocytes and macrophages, intranuclear inclusions in some cells and many syncytia formation (multinucleated giant cells), mainly in the cortical region (Fig. 6D), were observed in lymph nodes. The brain showed non-purulent encephalitis, edema, congestion, and areas of demyelination. Foci of gliosis were observed mainly located in the thalamus, hippocampus, midbrain, and cerebellum in the molecular layer of the folia. Changes in the Purkinje cells varied from mild degeneration of a few cells to widespread necrosis and depletion variably affecting different cerebellar folia (Fig. 6E). In these cases, immunohistochemistry often revealed proliferation of Bergmann glial cells (Fig. 6E), and empty spaces suggested the loss of many Purkinje cells. We also observed neural degeneration, necrosis and neuronophagia. Focal interstitial nephritis in the renal cortex with an inflammatory infiltrate mainly consisted of lymphocytes, macrophages, and syncytial cells was the main lesion in the kidney. We also observed tubulonephrosis with multifocal necrosis of tubular epithelial cells and associated karyorrhexis and karyolysis. Lungs showed congestion and edema. We did not observe relevant changes in other tissues. Immunohistochemistry revealed the CDV-antigen in endothelial cells of small blood vessels located in the kidney, adrenal glands (Fig. 6F), spleen and pancreas, as well as in the epithelial cells of renal collecting tubules and loop of Henle. qPCR molecular study identified CDV in paraffin embedded samples from kidney, however sequencing could not be performed due to poor quality RNA.

## Discussion

Understanding mortality causes is crucial for the conservation and management of endangered species, especially in those populations inhabiting anthropized landscapes where both natural and human-caused mortality may hinder the conservation of these species. This is particularly important for charismatic species commonly interacting with humans and their activities and for which these interactions may hinder their recovery, despite the investment of large amounts of money. Our study reveals that the main mortality causes of Cantabrian brown bears have changed since the population started to recover in the last decades. Although direct human persecution was prevalent during the last century [28], the importance of natural traumatic lesions and infectious diseases have recently increased and seem to be the main reasons behind brown bear deaths in the Cantabrian Mountains nowadays. Our results are in line with those from other small and isolated brown bear populations under recovery, such as Trentino (Northern Italy), where natural causes explain most brown bear mortality [29], but contrasts with larger European populations (e.g., Croatia, Slovenia), where human activities such as legal hunting, removal interventions or traffic collisions usually explain most bear deaths registered [30, 31]. However, in areas where hunting is legal that mortality might be compensatory, and those animals could have also died after suffering an infectious disease. In addition, causes of death may be biased towards deaths attributed directly to humans because, being non-threatened populations, necropsies are not performed on all animals nor are extra efforts invested in searching for bears’ cause of death.

### Relevance of infectious diseases

Our results suggest that infectious diseases are frequent in the Cantabrian brown bear population, particularly in some areas of the southwestern part of the population, where all the cases presenting primary infectious diseases were detected. The large prevalence of pathogens could be at least partially explained by a weak immune system in the population, a possible consequence of the isolation and the subsequent limited genetic diversity of the population [32]. The relevance of primary infectious diseases (i.e., infectious canine hepatitis, distemper, clostridiosis, or colibacillosis), which caused the death of 15.79% of the animals analyzed in this study, contrasts with other bear populations, where infectious diseases usually represent a marginal cause of death. For instance, in a previous study, only one out of 98 bear deaths analyzed in a Swedish population was linked to an infectious disease [33].

According to our findings, infections caused by *Clostridium* spp. bacteria such as *C. sordellii* or *C. novyi* (the latter described in ursids for the first time in this study) might be affecting a relevant fraction of the Cantabrian bear population (Table 1), since clostridia were found in 7 bears out of 38 whose death cause could be determined. Clostridia are anaerobic bacteria that proliferate under stressful situations, causing gangrenous myositis [34, 35]. All the cases but one reported in this study were associated either with traumatic injuries produced during intraspecific aggressions or in animals being trapped with snares. We believe that these stressful situations may have triggered ideal conditions for *Clostridium* spp. growth, ultimately causing the death of all these animals. Conversely, the bear 53 died in a traffic accident, although he showed previous clostridial disease, which on the other hand might have favored the accident due to abnormal behavior.

We detected colibacillosis in two Cantabrian brown bears. Although most *E. coli* serotypes are asymptomatic and common components of mammals’ intestinal microbiota, few foodborne strains such as VTEC are pathogenic, causing intestinal problems like bloody diarrhea that can be fatal for vertebrate species, including humans [36, 37]. The characteristics of the two bears dead due to *E. coli* in our study (i.e., a cub younger than one month and an adult female with severe traumatic injuries) would suggest that stressful situations and weak immune systems trigger the proliferation of the pathogenic strains of *E. coli*, in one of the cases confirmed as VTEC strain. To the best of our knowledge, this is the first study reporting VTEC in a free-ranging brown bear, although pathogenic *E. coli* is known to have caused the death of a captive polar bear (*Ursus maritimus*) cub and has also been detected in American black bears *Ursus americanus* [38, 39]. Among other factors, free-roaming domestic animals such as dogs and cats, common in the study area, are potential reservoirs of pathogenic *E. coli* that may facilitate the spread of these strains among wildlife species [36]. Thus, improved veterinary control of owned animals and targeted control of feral specimens, together with systematic wildlife health monitoring, are key management actions to minimize the risk of spreading pathogenic *E. coli* in the region.

In addition to bacterial infectious diseases, some viruses might also compromise the recovery of Cantabrian brown bears. For instance, we detected CDV and CAdV-1 in one and three bears, respectively (Table 1), all of them since 2014. Those viruses can be transmitted through direct contact with infected animals or contaminated fomites. Even when not directly lethal in most cases, infections by these viruses may promote neurological deteriorations and behavioral changes triggering traumatic injuries. For instance, we believe that the histopathological lesions detected in bear 47, caused by CDV disease, could prevent the animal from avoiding the traffic accident she likely suffered (i.e., due to neurological clinical signs), since she was found on the shoulder of a road.

Although fatal cases of CDV have previously been described in other bear species both in captivity and in the wild [40, 41], our work represents the first study suggesting CDV as the cause of death of a free-ranging brown bear. The CDV is a widely distributed *Morbillivirus* (a genus that includes several viruses that have caused devastating outbreaks in both animals and humans [42]), that affects domestic and wild mammals, primarily carnivores [43]. The detection of CDV antibodies in 12% and 37% of free-ranging brown bears in Slovakia and Central Italy [44, 45], respectively, suggests that CDV already circulates in several brown bear populations, at least in Europe. In the Cantabrian Mountains, CDV was previously detected in wild mustelids and canids, and it is responsible for the death of polecats *Mustela putorius*, badgers, martens and foxes since 2018, suggesting that the virus is already widespread in the region [43, 46]. These wildlife species, together with rural dogs, might be acting as sources of CDV infection for Cantabrian bears.

The current recovery of the Cantabrian bear population, together with the preservation of other wild carnivores and traditional livestock practices, will promote new inter and intraspecific interactions that may favor the transmission of all type of infectious diseases, including fatal ones. Thus, future research must analyze the actual effects of these and other infectious diseases on Cantabrian bear demography, as well as the factors that may enhance or limit their transmission. In line with [19], we also call for (1) standardized protocols of sample collection for both live and dead animals, (2) routine bear and other carnivores’ health monitoring, as well as for the (3) implementation of systematic health programs mainly in rural domestic animals. We strongly believe that these are useful tools to effectively detect the presence of infectious diseases in an animal and to prevent and act against epidemic outbreaks compromising the recovery of the Cantabrian bear population.

### Intraspecific aggressions

Most Cantabrian bear deaths registered were associated with traumatisms caused during intraspecific agonistic interactions (e.g., adult-adult fights or infanticides). These aggressions are common in the species, especially during mating season (late spring and early summer) when adult males try to kill cubs to obtain a mating opportunity with the victimized female, which usually defends her cubs aggressively from these attacks [47]. Recent records in the Cantabrian Mountains confirm the death of cubs and adults of both sexes during these interactions [19]. Variables such as habitat fragmentation, small forest coverage and high human density may prone infanticide by male brown bears [47]. Although females with cubs try to settle in the roughest areas of the Cantabrian Mountains, some still frequent the same areas than adult males, increasing the probability of risky encounters [48]. According to [20], around 40% of Cantabrian brown bears die during their two first years of life, numbers that are similar to those reported in other populations [49]. In this context, direct observations registered by the staff of the Brown Bear Foundation, a non-governmental organization (NGO) working in the Cantabrian Mountains, suggest that infanticides may explain up to 80% of the total cub mortality in the area, especially in the first months after den abandonment, coinciding with the mating season [19, 50]. Although the lack of infanticide reports outside the mating season in the area supports the idea that other factors such as food shortage, known to act in other populations with larger cub mortality rates [51], may be negligible in the Cantabrian population [19], all the information registered suggests that sexually selected infanticide, which might be prompted by increasing human activity in recent years, is likely to affect Cantabrian bear demography [47]. In this context, a proactive conservation of the roughest areas, especially those close to human settlements and outdoor human activities potentially forcing the displacement of females with cubs to more risky habitats, has already been proposed as a key management action to minimize the effects of sexually selected infanticide in the recovery of Cantabrian bears [48]. Therefore, and considering that infanticides seem to be especially frequent in small and isolated populations [47], analyzing possible changes in the prevalence of infanticidal events under the current context of the recovery of the population must be a key aspect for the management of Cantabrian bears.

### Human-caused mortality

The relevance of direct human-caused mortality of Cantabrian bears may have decreased since the population reached its historical low during the end of the last century, when direct persecution by humans caused most of the reported deaths [19, 20]. For example, at least 29 brown bears were poached in the region between 1982 and 1994 [20]. Although intentional poaching and poisoning still occur in the region, their intensity seems to have decreased in the last decade. We believe that the increasing interest for bear-related ecotourism, which has become a popular activity in the area in the last decade, might also explain the decreasing levels of poaching reported in the region. In addition, other human-caused mortality causes commonly reported in several brown bear populations also appear to be less relevant in the Cantabrian population. For instance, while collisions with vehicles are a common cause of bear deaths in several European and American bear populations [30–32, 52], they seem to be much less frequent in the Cantabrian Mountains (10.53% in our study). Abrupt landscapes and winding local roads within Cantabrian bear range, which prevents driving at high speeds, may explain the relatively low numbers of brown bears involved in traffic accidents in the region; however, sustained population growth observed in recent years may favor traffic accidents in the future [33].

### Final remarks

Our findings suggest that the main mortality causes of Cantabrian bears have changed since the population started to recover. We are aware that our dataset only represents a minor percentage of all brown bears dead during the study period considered, especially when we refer to adults that are much more difficult to track due to their huge home ranges, the difficult accessibility of some areas and the lack of telemetry data. Despite our small sample size, which is a consequence of the small population size and the already mentioned scarcity of telemetry data, we are confident that our findings represent a good reflection of what is happening in the area, especially in the last decade, which includes more than 80% of all the bears registered. Although our data do not allow us to calculate mortality rates and their impact on brown bear demography, we believe that the mortality causes we report here are a good indicator of the main threats that Cantabrian bears currently face and that this knowledge is very useful to optimize the management actions carried out by the different administrations. Yet, we cannot neglect that common mortality causes in the present such as infectious diseases or traumatic injuries might be underrepresented during the beginning of the population’s recovery, when field observations of brown bears were much scarcer. In addition, the increasing prevalence of natural deaths and infectious diseases reported in recent years may also be a direct consequence of the better laboratory techniques used during the most recent necropsies [13].

The current recovery of the Cantabrian brown bear population implies not only an increase in bear numbers but also the colonization of new areas by the species and the emergence of new mortality causes such as those reported in this study. In this new scenario, legal instruments for the conservation of the population, which need to be urgently updated [18], must include new regulations and investments in measures aiming at mitigating not only the main mortality causes described in this study, but also those expected to be more frequent in the near future (e.g., an increase in the frequency of traffic accidents as a consequence of the colonization of sub-optimal areas). To increase the tolerance and to maintain the low levels of direct human persecution toward the species, these management actions must also include intensive field monitoring and expand some existing measures such as the prevention and compensation schemes to the entire current Cantabrian bear range. In addition to all this, more research about the real impacts of infectious diseases in Cantabrian bear demography and the possible strategies to minimize the prevalence of these diseases is urgently needed.

### Supplementary Information

**Additional file 1. Presence of birefringent crystals compatible with calcium oxalate (arrows) in kidney from bear 36 (A and B). **The images show the advanced state of autolysis with total loss of the kidney structure. Hematoxylin and eosin stain, using polarized light.

**Additional file 2. Pathological findings in brown bear 49 showing clostridial gangrenous myositis due to *Clostridium novyi*. A** Left ribs from the 7^th^ to the 13^th^. Fractures are shown. **B** Left 11^th^ rib. Clostridial-like bacilli are observed. Gram stain. **C** Longissimus dorsi skeletal muscle. Serohemorrhagic edema, emphysema and hemorrhages are observed, as well as thrombi formed by bacilli in the lumen of one vessel. Hematoxylin and eosin stain. **D** Longissimus dorsi skeletal muscle. Numerous Gram positive clostridial-like bacilli are present within the vessel. Note that bacteria are invading the endothelium (inset). Gram stain.

## Data Availability

The data supporting the findings of this study are available by the corresponding author upon reasonable request.
