# Supplementary figures and images for: New threats in the recovery of large carnivores inhabiting human-modified landscapes: the case of the Cantabrian brown bear (Ursus arctos)
Source: Vet Res. 2024 Feb 23;55:24. doi: 10.1186/s13567-024-01279-w (PMC10893660; doi:10.1186/s13567-024-01279-w)

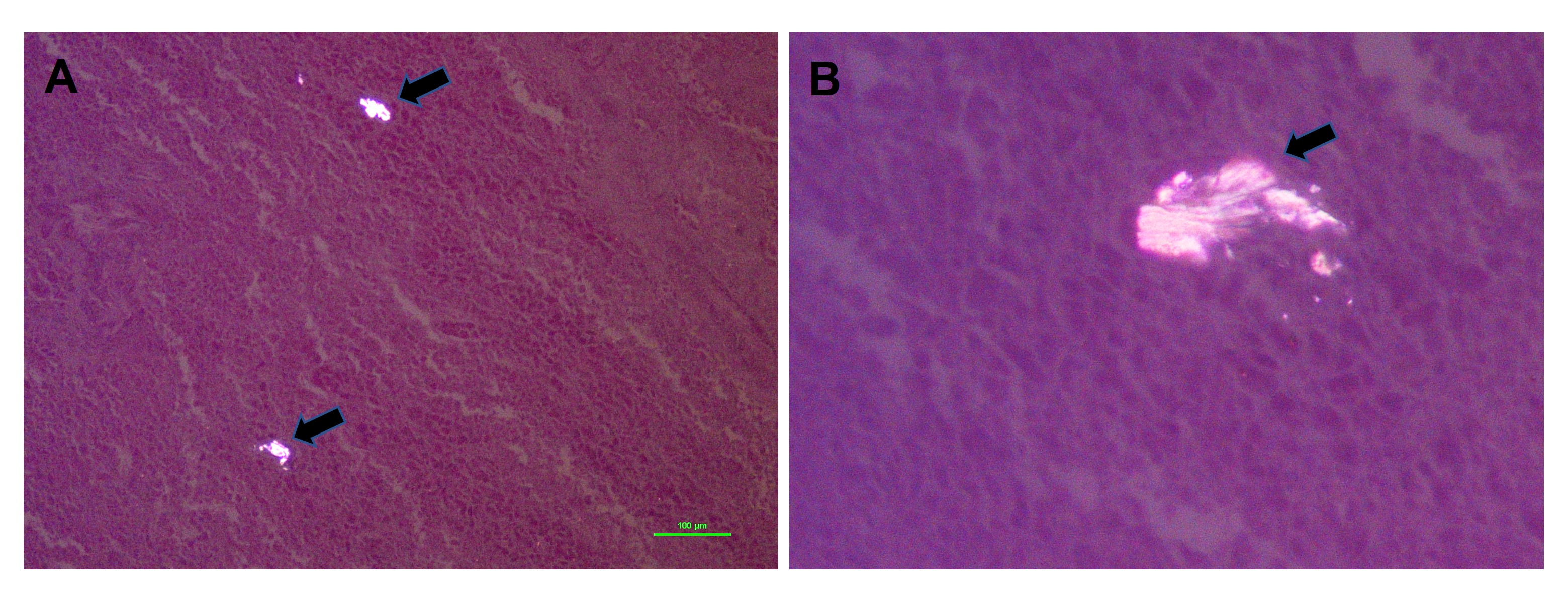

Supplement: Supplementary file 1 — Additional file 1. Presence of birefringent crystals compatible with calcium oxalate (arrows) in kidney from bear 36 (A and B). The images show the advanced state of autolysis with total loss of the kidney structure. Hematoxylin and eosin stain, using polarized light. [file 13567_2024_1279_MOESM1_ESM.jpg]

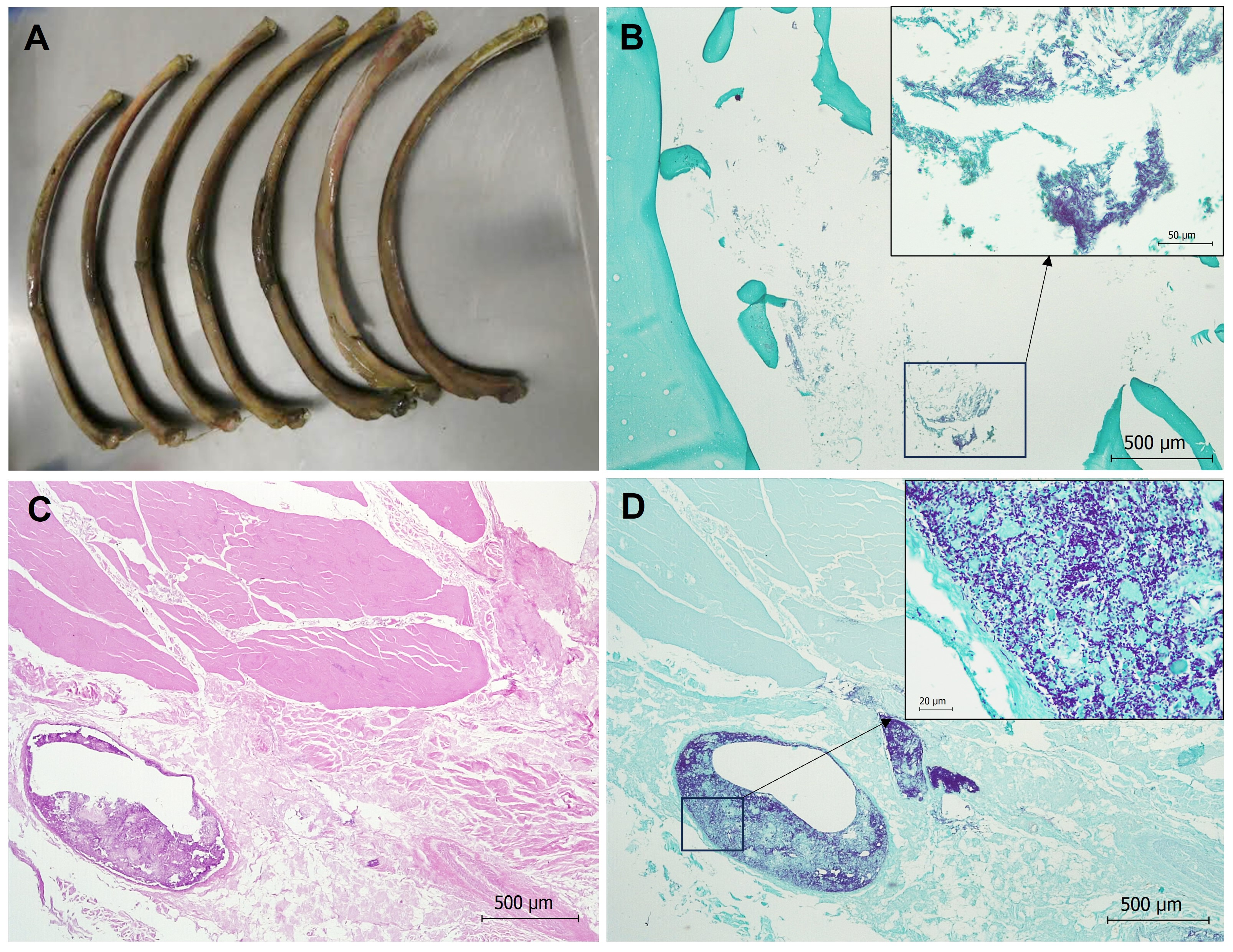

Supplement: Supplementary file 2 — Additional file 2. Pathological findings in brown bear 49 showing clostridial gangrenous myositis due to Clostridium novyi. A Left ribs from the 7th to the 13th. Fractures are shown. B Left 11th rib. Clostridial-like bacilli are observed. Gram stain. C Longissimus dorsi skeletal muscle. Serohemorrhagic edema, emphysema and hemorrhages are observed, as well as thrombi formed by bacilli in the lumen of one vessel. Hematoxylin and eosin stain. D Longissimus dorsi skeletal muscle. Numerous Gram positive clostridial-like bacilli are present within the vessel. Note that bacteria are invading the endothelium (inset). Gram stain. [file 13567_2024_1279_MOESM2_ESM.jpg]
